# Climate, not grazing, influences soil microbial diversity through changes in vegetation and abiotic factors on geographical patterns in the Eurasian steppe

**DOI:** 10.3389/fpls.2023.1238077

**Published:** 2023-09-06

**Authors:** Bademu Qiqige, Bin Wei, Yuqi Wei, Mohan Liu, Yixian Bi, Ruixuan Xu, Nan Liu, Gaowen Yang, Yingjun Zhang

**Affiliations:** ^1^ Department of Grassland Science, College of Grassland Science & Technology, China Agricultural University, Beijing, China; ^2^ Key Laboratory of Grasslands Management and Utilization, Ministry of Agriculture and Rural Affairs, Beijing, China

**Keywords:** climate factors, grazing intensity, soil microbe, vegetation, soil physicochemical properties

## Abstract

Livestock grazing has a significant impact on the biodiversity of nature grassland ecosystems, which is mainly regulated by climate factors. Soil microbes are essential components of biogeochemical cycles. However, the coupling effects of grazing with MAT (mean annual temperature) and MAP (mean annual precipitation) on soil microbial communities remain inconsistent. Our study considered the various climates in four grasslands as natural temperature and precipitation gradients combined with grazing intensity (GI). We collected and analyzed vegetation and soil physiochemical properties from four grasslands. Our results showed that climate factors (CF) changed β diversity of soil bacteria and fungi while grazing intensity and their interaction merely affected fungi β diversity. Furthermore, climate factors and grazing intensity impacted changes in vegetation and soil physiochemical properties, with their interaction leading to changes in EC and MBC. Our analysis revealed that climate factors contributed 13.1% to bacteria community variation while grazing intensity contributed 3.01% to fungi community variation. Piecewise SEM analysis demonstrated that MAT and MAP were essential predictors of bacteria β diversity, which was significantly affected by vegetation and soil carbon and nitrogen. At the same time, MAP was an essential factor of fungi β diversity and was mainly affected by soil nitrogen. Our study indicated that bacteria and fungi β diversity was affected by different environmental processes and can adapt to specific grazing intensities over time.

## 1 Introduction

Grasslands are a significant resource that accounts for nearly 40% (59 million km^2^) of the global ice-free land area, and have multiple functions in the ecology system and service (Hufkens et al., 2016; Hautier et al., 2018). Different climates are the primary reason for various grasslands, profoundly affecting grassland ecosystems by changing precipitation patterns, increasing temperatures, and regulating the entire ecosystem processes (Berdugo et al., 2020; Hu et al., 2022). Livestock grazing provides essential survival supplements and is vital for herder’s livelihood and maintaining multiple ecosystem functions. Anthropogenic disturbances such as grazing and fire, connected with global climate change, may have complex effects on grasslands (Jing et al., 2015). Most of our earth is undergoing climate warming, changing precipitation patterns, and increased extreme climate events (Showstack, 2013; Hoover et al., 2022). These phenomena have significantly impacted terrestrial ecosystems, including altering vegetation productivity. Previous researches have examined the influence of changing temperature and precipitation on grassland ecosystems, while still existed inconclusive results (Nolan et al., 2018; Berdugo et al., 2020). Experiments by Ma et al. (2017) in Tibetan Plateau found that precipitation changes did not affect biomass levels. At the same time, climate warming reduced the temporal stability of biomass, primarily affecting dominant species related to community biomass stability instead of plant species diversity, such as S*tipa aliena* and *Elymusnutans* (Ma et al., 2017). Therefore, it is crucial to consider the context-dependent of climate change or precipitation-altering plant community biomass or biodiversity, which is closely linked with the local environment and their grassland ecosystem types (Gherardi and Sala, 2019; Liu et al., 2021). Additionally, aboveground primary productivity (ANPP) was highly sensitive in arid sites and primarily limited by precipitation in local places. Meanwhile, plant growth is limited by other resources, such as nutrients, light, and temperature. However, a recent global meta-analysis found that the vegetation Shannon index H and species richness was not significantly affected by climate warming, even showing negative relationships under drought, while they were positively related to local MAP (Liu et al., 2021).

Climate change also significantly impacts soil physicochemical properties through vegetation (as intermediation), with plant allocation of carbon to their above- and below-ground organs constituting terrestrial carbon dynamics. The quantity and quality of carbon input are closely associated with plant types, which are primarily limited by climatic conditions and their interactions with soil environments (Henneron et al., 2020; Mekonnen et al., 2021). The climate is considered the dominant factor in controlling soil carbon pool dynamics on global or regional scales, inextricably linked with nutrient cycling and soil microbial communities, which have a significant impact on the biogeochemical cycling of macronutrients, micronutrients and other primary elements essential for the growth of plant and animal (Bardgett and van der Putten, 2014; Schimel, 2016). Recent research has also highlighted how climate changes affected soil microbial communities, with direct effects tracing back as far as 1,000 years ago, with paleoclimate imprinting on contemporary soil bacteria (Fordham et al., 2017; Liu et al., 2023; Luo et al., 2019). Climate change also strongly affects bacterial richness and community through pH, soil properties, and soil organic carbon concentration in forest ecosystems on a global scale (Looby and Treseder, 2018; Zheng et al., 2020). However, the impacts of climate change on soil microbial communities remain understudied in grassland systems.

Grazing is a precious utility pattern for grasslands, providing humanity with economic growth through low investment, high returns, and ecosystem stability (Bai et al., 2004). However, while herbivores can change plant biomass and biodiversity, soil biogeochemistry, and microbial communities through activities such as defoliation, trampling, and returning dung and urine, grazing effects on the soil microbial community still need to be determined to technical and methodological constraints. The advent of high-throughput sequencing and metagenomics is instrumental in demonstrating microbial diversity and composition in various soil ecosystems, providing practical approaches for different ecosystem responses to various climate change scenarios. Over the past 15 years, numerous studies have explored the influence of grazing on grasslands, which was controversial globally. Different grazing intensities can change aboveground plant biomass directly, and moderate grazing can maintain grassland system diversity. The feeding preferences of herbivores alter the dominant vegetation species in grassland communities (Koerner et al., 2018). Grassland biomes store 10%–30% of global soil organic carbon, and grazing and global climate change are essential factors in grassland carbon dynamics and the global carbon process (Follett and Reed, 2010; Zhou et al., 2019). Meta-analyses showed that soil carbon content increased under light grazing in the subsoil layer (>20 cm), while it decreased on the topsoil under moderate and heavy grazing. Grazing also reduced soil carbon and nitrogen pools in grassland ecosystems and decreased soil microbial biomass carbon and nitrogen (21.62% and 24.40%) (Jiang et al., 2020; Zhang et al., 2018). Soil microbial communities play pivotal roles in ecosystem feedback under global climate change, extensively researched in recent decades. However, their interactions with human activities, such as grazing on grassland ecosystems, are poorly understood. Warming increased some proportion of oligotrophic lineages; However, it reduced those of active saprotrophic fungi and arbuscular mycorrhizae, and no interaction between warming change and grazing for soil fungi communities (Che et al., 2019). In Tibetan alpine meadows, microbial functional genes are tremendously altered by the interaction of grazing and warming rather than their individual effects (Jiang et al., 2020). While grazing has asynchronous influences on soil microbial community, increasing top and deep soil bacterial diversity, it does not significantly affect deep soil fungal diversity (Wu et al., 2022).

Understanding and predicting the influences of climate factors and grazing intensity on soil microbial community is a grand challenge in our grassland ecosystems. Our study aimed to answer three critical issues through field experiments in the northwest grasslands of China: firstly, which factors contribute more significantly to the soil microbial community? How does soil microbial diversity respond to climate factors and grazing intensity? Moreover, what is the influence of vegetation and soil physiochemical properties on the soil microbial community? We hypothesized that climate factors influence soil microbial community more than grazing intensity on a large spatial scale. Both climate factors and grazing affect soil microbial biodiversity independently without interaction effects. Climate factors and grazing intensity also changed microbial communities via vegetation and soil physiochemical properties.

## 2 Materials and methods

### 2.1 Study sites

**Figure 1:**
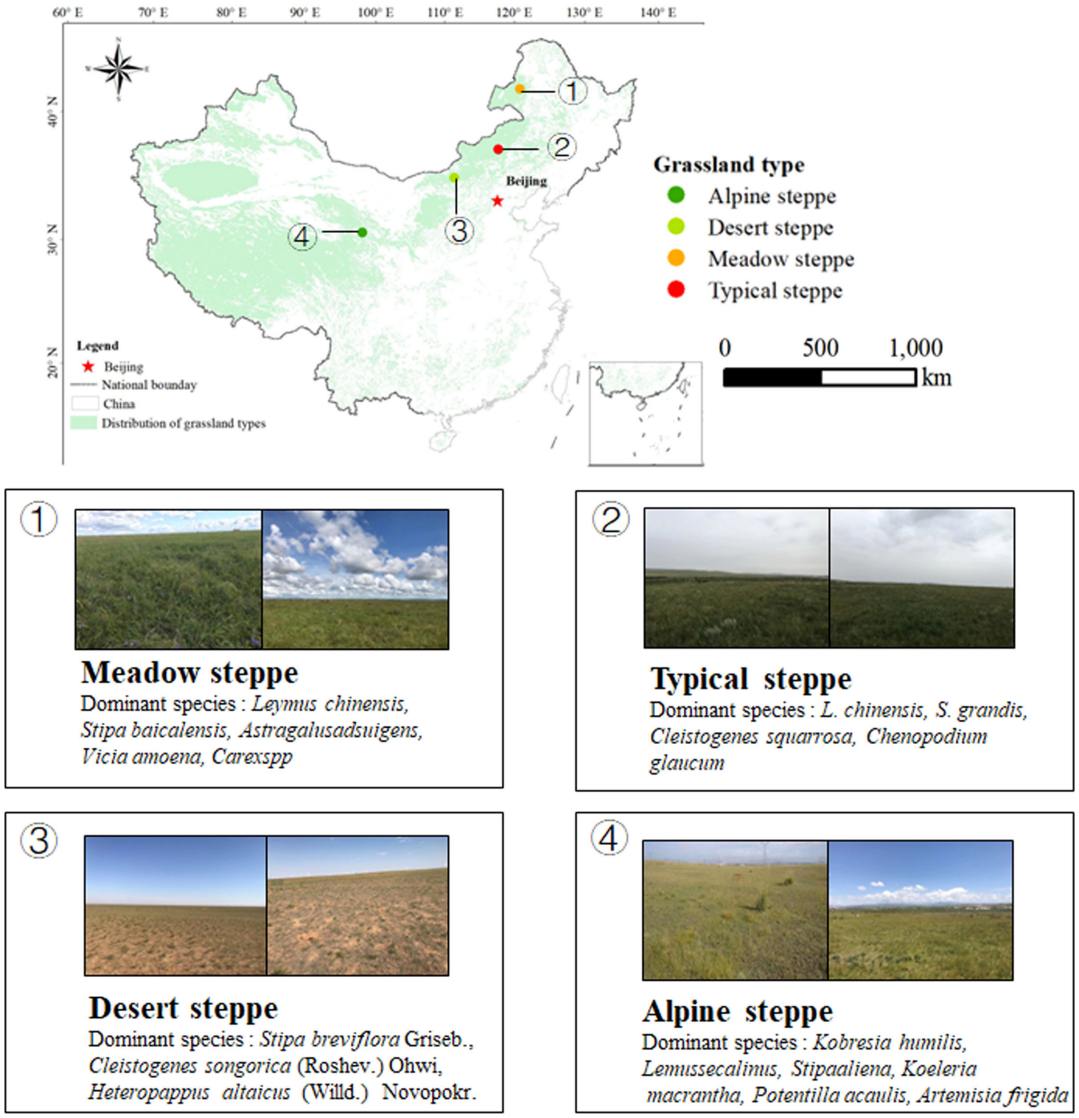
The location of the sampling sites.

We carry out our study in four sites in northern China, from Inner Mongolia to Qinghai-Tibetan Plateau; Meadow steppe (MS, lat. 49°19′ N, long. 119°56′ E, 666-680 m) is located in Hulunbeer, the average annual precipitation is 375 mm and the annual average temperature is -3.5 °C, which is dominated by *Leymus chinensis*, *Stipa baicalensis*, soil type of it is chernozem; Typical steppe (TS, lat. 44°15′ N, long. 116°32′, 1111-1121 m) is located in Xilinhaote, the average annual precipitation is 350 mm and the annual average temperature is -0.1 °C, which is dominated by *L. chinensis, S. grandis*, soil type of it is chestnut; Desert steppe (DS, lat. 41°56′ N, long. 111°09′, 1000-1031 m) is located in Suniteyouqi, the average annual precipitation is 177.2 mm and the annual average temperature is 4.4 °C, which is dominated by *Stipa breviflora* Griseb., *Cleistogenes songorica* (Roshev.), soil type of it is brown carcic soil; Alpine steppe (AS, lat. 36°92′ N, long. 100°93′, 3200) is located in Xihai Town, Qinghai-Tibetan Plateau, the average annual precipitation is 424.82 mm and the annual average temperature is 1.4 °C, which is dominated by *Kobresia humilis, Lemus secalinus*, soil type of it is clay loam. **Figure 1** shows the coverage of the four grassland types in the region.

### 2.2 Experimental design

Study platforms were established and set different grazing intensities in 2008 (MS), 2010 (DS), 2014 (TS), and 2018 (AS) respectively. Grazing intensity was reasonably distributed across four gradients, namely, CK, Light grazing (LG), Moderate grazing (MG), and Heavy grazing (HG) in four grassland types, considering grassland productivity, herd types, and areas. Grazing periods were limited to June to October, with no grazing allowed during the rest of the year. The grazing experiments followed a randomized block design. We investigated and collected vegetation and soil samples in these four sites in August 2019.

### 2.3 Measurement of vegetation and edaphic properties

The plant biodiversity and biomass were assessed by randomly selecting three 0.5m×0.5m quadrats in each plot in four sites. The samples were then classified according to the species composition, and the biomass was recorded before drying them into an oven at 65°C (48 h). The soil properties were determined using three cylindrical soil cores collected from where above-ground biomass had been sampled. Soil samples were homogenized, sieved, and divided into three subsamples, each passing through a 2 mm sieve. The first subsample was under air-dried to measuring the physicochemical properties; The second subsample was reserved at 4°C and immediately analyzed soil ammonium (NH_4_^+^-N) and nitrate (NO_3_^-^-N) contents, soil microbial biomass carbon C (MBC), and soil microbial biomass carbon N (MBN); The third subsample was stored at -80°C for DNA analysis.

Soil parameters were analyzed as follows: pH of soil was measured using a deionized, and water soil ratio of 2.5:1; Soil water content (SWC) was measured by gravimetric method; Elemental analysis (Elementar Analysensysteme GmbH) measured Soil organic carbon (SOC); Elemental Analyzer (vario MACRO cube, Elementar, Germany) measured total nitrogen (TN); Soil inorganic nitrogen was extracted with a 2M KCl solution used to extract soil ammonium (NH_4_^+^-N) and nitrate (NO_3_^-^-N), then analyzed with a FIAstar 5000 (FOSS Analytical) (Pang et al., 2020); Olsen method measured available phosphorus (AP) content; Two 10 g fresh soil subsamples were used to measure the MBC and MBN contents using the chloroform fumigation extraction method, one of which was fumigated with chloroform for 24 h, and the other was not fumigated. After finishing fumigation, both soil subsamples were extracted with 25 ml 0.5 M K_2_SO_4_ on a shaker for 30 min (Vance et al., 1987). The extracts were filtered and then measured by a multi C/N analyzer (Multi N/C3100, Analytik Jena AG, Germany).

### 2.4 Soil DNA extraction and PCR analyses

To extract whole microbial genome DNA from the soil samples, three replicates of 0.5g of soil were used and processed by the FastDNA SPIN for soil kit (MP Biomedicals, Solon, USA) based on the instructions of manufacturer. 1.0% agarose gel electrophoresis used to quantify DNA and a NanoDrop® ND-2000 spectrophotometer (Thermo Scientific Inc., USA) before being stored at -80°C. The bacterial 16S rRNA (V3-V4) and fungal ITS genes (ITS1F_ITS2R) were amplified by primers pairs 338F (5’-ACTCCTACGGGAGGCAGCAG-3’) and 806R (5’-GGACTACHVGGGTWTCTAAT-3’) for bacteria and primers pairs (ITS1F: 5’-CTTGGTCATTTAGAGGAAGTAA-3’ and ITS2R: 5’-GCTGCGTTCTTCATCG ATGC-3’) for fungi (Liu et al., 2016). The PCR reactions included 4 μL 5× Fast Pfu buffer, 2 μL 2.5 mM dNTPs, 0.8 μL of each forward and reverse primers (5 μM), 0.4 μL Fast Pfu polymerase, 10 ng of template DNA, and ddH_2_O to a final volume of 20 µL. PCR amplification cycling conditions constituted of initial denaturation at 95 °C for 3 min, and following by 27 cycles of denaturing at 95 °C for 30 s, annealing at 55 °C for 30 s, and extending at 72 °C for 45 s, and a single extension at 72 °C for 10 min, following by a final step at 4 °C. The PCR product was using the AxyPrep DNA Gel Extraction Kit (Axygen Biosciences, Union City, CA, USA) and quantifying by the Quantus™ Fluorometer (Promega, USA). It was then sequenced on the Illumina MiSeq PE300 platform/NovaSeq PE250 platform (Illumina, San Diego, USA) according by Majorbio Bio-Pharm Technology Co. Ltd (Shanghai, China).

### 2.5 Statistical analyses

Our data were statistical analyses by R 4.2.1 (R Development Core Team, 2022). Two-way ANOVA was performed to analyzing the soil microbes community, soil physicochemical properties, plant communities, and climate factors (MAP and MAT), grazing intensity, and their interactions as fixed factors. The connection between plant, soil properties and soil microbe communities were assessed by redundancy analysis (RDA). The contribution percentage and significance of climate factors, grazing intensity, soil, and vegetation to the soil bacterial and fungal community were calculated by the rdacca.hp package, and visualized by the UpSetVP package to show hierarchical segmentation. The association between soil property (e.g. TN, SOC, NO_3_^-^-N, NH_4_^+^-N) and bacterial or fungal β diversity was analyzed using PicewiseSEM after accounting for multiple key ecosystem factors such as Microbes (MBC, MBN), plant (richness, Shannon, AGB, BGB) (Lefcheck, 2016). All measured variables were considered in this model and analyzed as composite variables using the “piecewiseSEM” and “lme4” packages (Bates et al., 2014). Fisher’s C test confirmed correctness of the results, and the models were modified according to the significance value (*P* < 0.05).

## 3 Results

### 3.1 The effect of climate factors and grazing intensity on microbes

**Figure 2:**
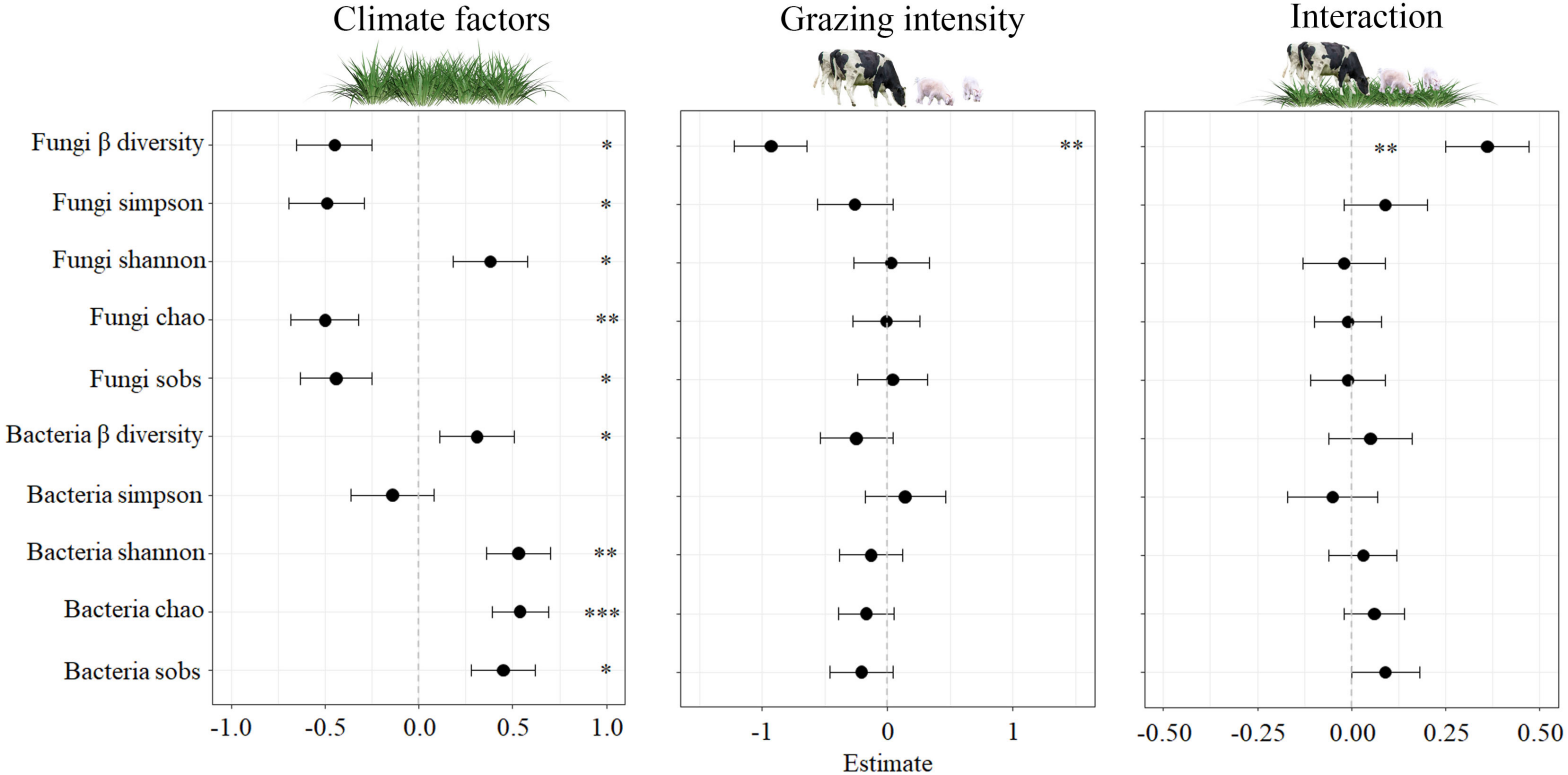
Effects of climatic factors, grazing intensity and their interaction on standardised soil microbes for the four study sites. The significant effects (*0.01 < *P* < 0.05; **0.001 < *P* < 0.01; *** *P* < 0.001) are labelled with * Fungi β-diversity, fungi Simpson, fungi Shannon, fungi chao, fungi forbs, bacteria β-diversity, bacteria Simpson, bacteria Shannon, bacteria chao, bacteria sobs.

Our study examined impacts of climate factors (CF) and grazing intensity (GI) on soil microbe diversity and the interaction between the two. As shown in **Figure 2**, CF had a significant effect on many measures, including fungi β diversity, fungi Simpson, fungi Shannon, fungi sobs, bacteria β diversity, bacteria sobs (*P* < 0.05), fungi chao, bacteria Shannon (*P* < 0.01), and bacteria chao (*P* < 0.001). However, GI and the interaction between CF and GI did not significantly impact microbe diversity, except for fungi β diversity (*P* < 0.01). Labels in **Figure 2** indicate the significant effects observed.

### 3.2 The interaction of climate factors and grazing intensity for plant and soil property

**Figure 3:**
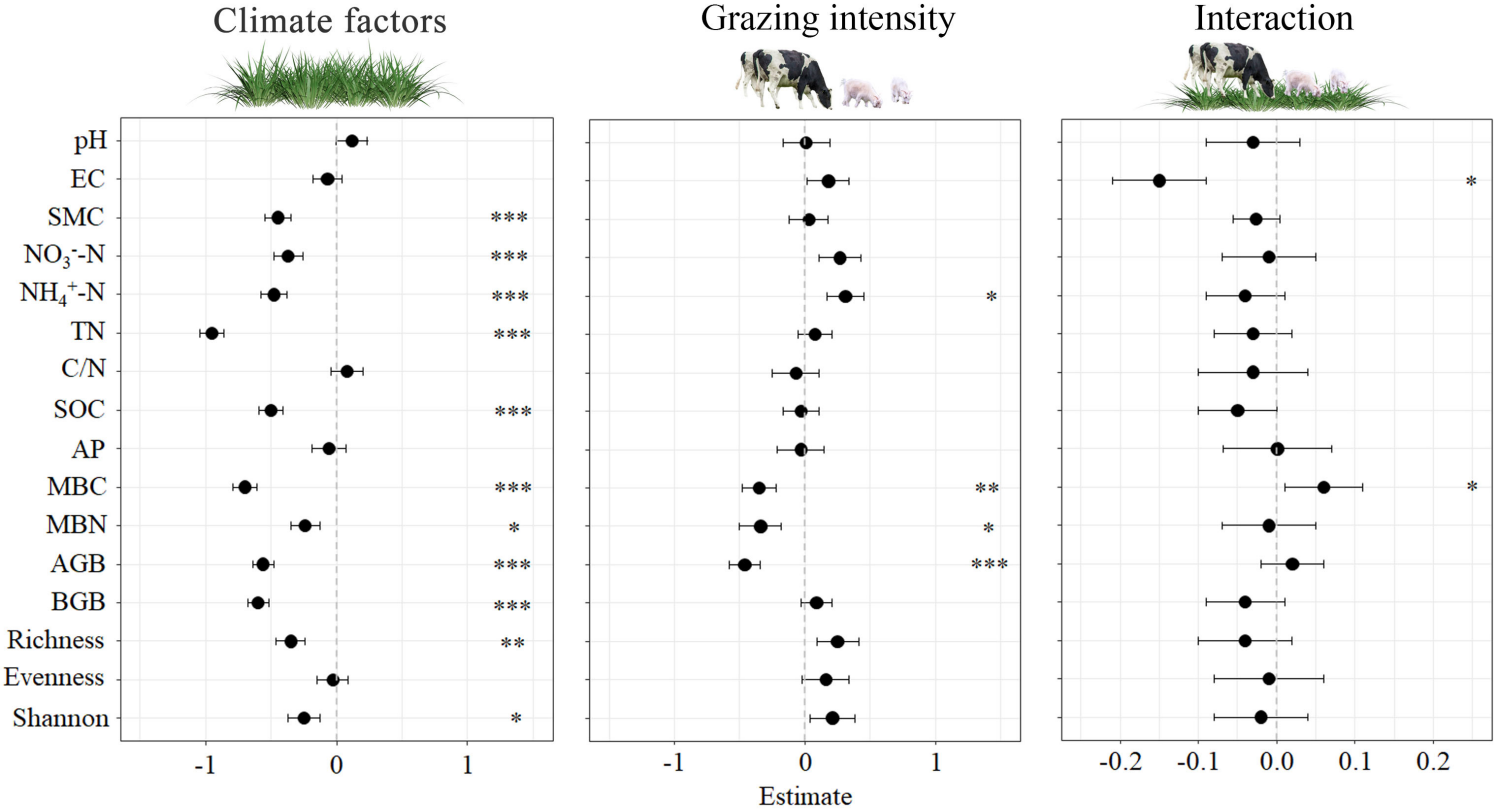
Effects of climatic factors, grazing intensity and their interaction on standardisation soil and vegetation parameters for the four studied sites. Significant effects (*0.01 < *P* < 0.05; **0.001 < *P* < 0.01; *** *P* < 0.001) are indicated by * pH, EC, SMC, NO_3_^-^-N, NH_4_^+^-N, TN, C/N, SOC, AP, MBC, MBN, plant AGB and plant BGB, plant richness, evenness and Shannon.

Our study examined impacts of grazing intensity and climate factors on plant community and soil physicochemical properties, as well as the interaction between the two. As presented in **Figure 3**, both climate factors and grazing intensity had significant effects on plant Shannon, soil MBN (*P* < 0.05), plant richness (*P* < 0.01), SMC, NO_3_^-^-N, NH_4_^+^-N, TN, SOC, MBC, plant AGB and plant BGB (*P* < 0.001). However, pH, EC, and plant evenness (*P* > 0.05) were no significant effects. Grazing intensity significantly affected on NH_4_^+^-N, MBN (*P* < 0.05), MBC (*P* < 0.01), and AGB (*P* < 0.001), while pH, EC, SMC, NO_3_^-^-N, TN, C/N, SOC, AP, BGB, plant richness, plant evenness, and plant shannon index have not significant effects. The only significant responses observed for CF*GI were for EC and MBC. Labels in **Figure 3** indicate the significant effects observed.

### 3.3 Contributions of plant and soil indicators for β diversity of microbes

**Figure 4:**
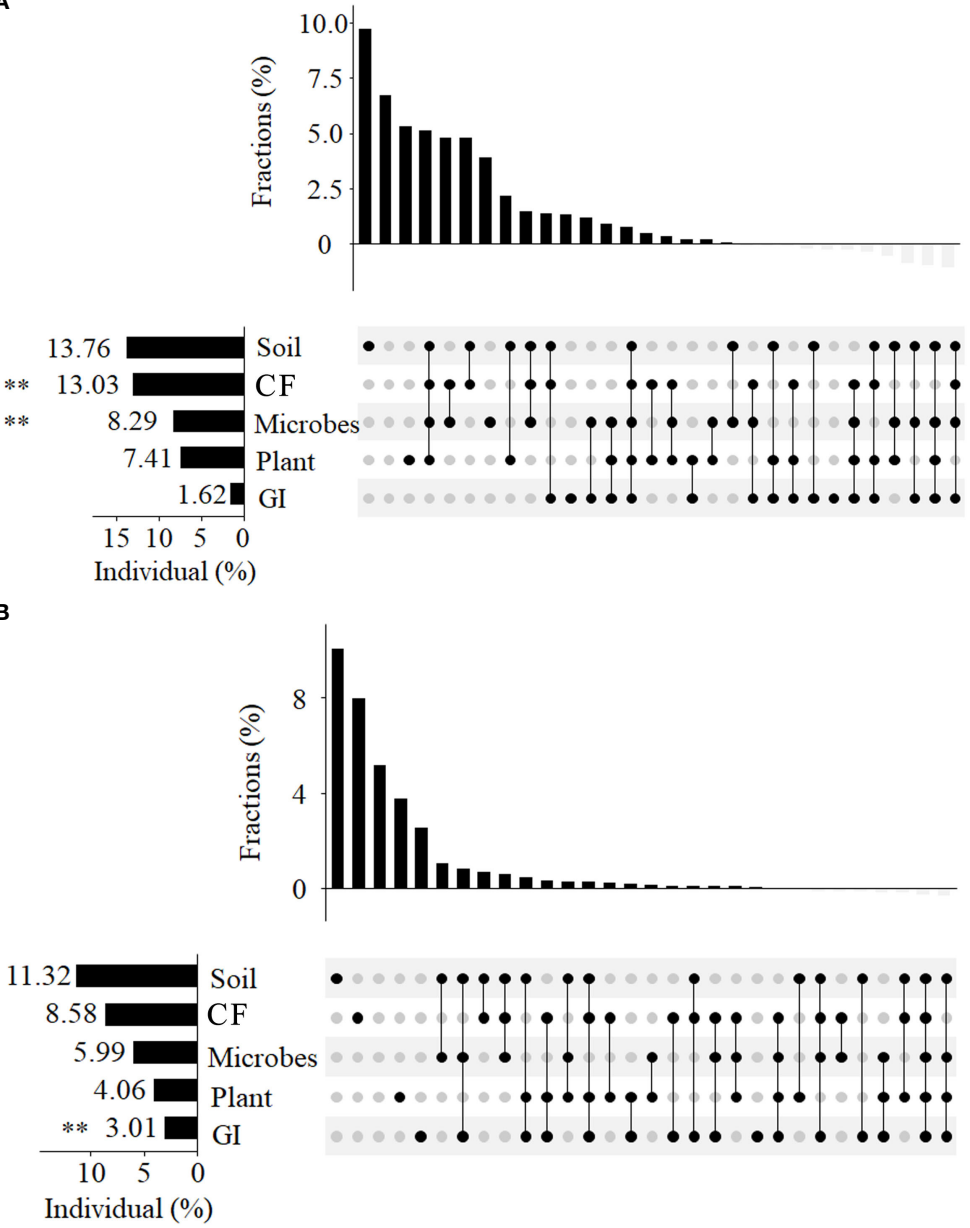
We utilized UpSetView variation-partitioning to analyze pure and shared contributions of soil properties, CF, microbes (microbial carbon and nitrogen), plant, and GI to variations in soil bacteria **(A)** and fungal **(B)** communities. The graphs depict the percentage of variance explained by the environmental factors, with dot matrices and associated bars above showing the values of exclusive and shared contributions. Negative values resulting from adjustment of R-squared were not included in the graph, but were incorporated in the total contribution of each variation, as indicated on the edge of the dot matrix. The significant effects *0.01 <*P* < 0.05; **0.001 < *P* < 0.01; *** *P* < 0.001.

Our study explored the contributions of biotic (plants and microbial communities) and abiotic factors (soil, CF, GI) to changes in bacteria and fungi β diversity, with varying degrees of influence observed (**Figure 4**). Of the explanatory factors, the soil had the highest contribution rate (13.76%), with significant contributions, also observed for GF and microbes (*P* < 0.01). Conversely, the contribution rates of plant and GI for β diversity were relatively low (7.41% and 1.62%, respectively). Some factors were found to commonly explain β diversity, with soil, CF, microbes, and plant accounting for 5.2%, CF and microbes for 5%, and soil and CF for 5.1%. Regarding fungi β diversity, both biotic and abiotic indicators have had significant impacts, with soil having the highest contribution rate (11.32%), followed by CF (8.58%), microbes (5.99%), and plant (4.06%). Grazing intensity significantly affected fungi β diversity, with soil and microbes commonly explaining its variation, along with soil, microbes, and GI.

### 3.4 The response of microbes β diversity to biotic and abiotic in various grazing types

**Figure 5:**
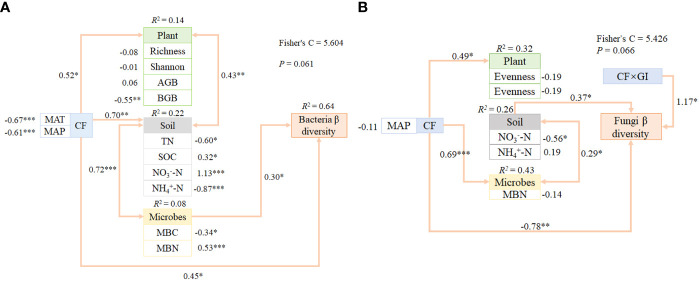
We utilized PiecewiseSEM to investigate the pathways through which climate factors and grazing intensity impacted soil bacteria and fungi communities via plant and soil properties at a large spatial scale. Composite variables were created for the soil properties, plant, and microbes. The signed path coefficients shown on the arrows represent the standardized effect size of the relationship, with non-significant relationships among residual variables of measured predictors not included. The significant effects (*0.01 <*P* < 0.05; **0.001 < *P* < 0.01; *** *P* < 0.001). Our results showed that the responses of soil bacteria β diversity **(A)** and fungi β diversity **(B)** were impacted by multiple direct and indirect effects of climate factors, grazing intensity, plant and soil properties. Pathways linking these variables were identified and the strength of their relationships were established, providing insight into the mechanisms that shape microbial community structure in grassland ecosystems.

We utilized PiecewiseSEM to examine the direct and indirect pathways through which regulatory factors impacted climate factors in microbes β diversity (**Figure 5**). Our results revealed that vegetation characteristics and soil physicochemical properties collectively played a significant role in explaining climate factors in both bacteria and fungi β diversity. Additionally, soil physicochemical properties, including climate factors (MAT, MAP), consistently demonstrated significant regulation of microbes β diversity by directly and indirectly altering SOC, NO_3_^-^-N, MBN, BGB, TN, NH_4_^+^-N, and MBC. MAT and MAP were important predictors of bacteria β diversity among climate factors. In contrast, factors such as soil properties, CF*GI, and CF collectively explained variability in fungi β diversity. CF*GI and MAP were found to impact fungi directly β diversity, with additional indirect effects on microbes that altered soil properties (e.g. NO_3_^-^-N and NH_4_^+^-N).

## 4 Discussion

### 4.1 Interactive effects of climate factors and grazing on soil microbes

Soil microbes play a crucial role in determining fitness and health of grassland and regulating ecological system. Microbial diversity, both alpha diversity which explores the structure or composition of microbial communities in a single habitat or treatment, and beta diversity, which represents the combination of microbial communities with environmental gradients, are commonly used to measure microbial communities (Gülay and Smets, 2015; Luo et al., 2018; Zhang et al., 2016). Our studies found the α diversity and β diversity of soil bacteria and fungi respond significantly to climate factors.

Warming has shown inconsistent effects on soil microbes in many studies. In Tibetan grasslands, warming after six years have significantly altered soil fungal communities. However, three-years of warming did not alter soil fungi communities. Seven years of warming did not significantly affect bacteria diversity, but it changed the fungi and bacteria communities. Therefore, the influence of climate for soil microbes depended on time of warming (Jiang et al., 2021). Similarly, warming changed α diversity of soil bacteria in the first year at temperate steppe of Inner Mongolia. However, there were no significant changes in the next two years. This suggests that the bacterial community is sensitive and adapted to warming, and increased the stability and complexity of the bacterial network structure (Ibarra et al., 2020). In contrast, a meta-analysis on a global scale has shown that warming increases microbial populations but decreases biodiversity (Guo et al., 2019). Besides warming, precipitation is also a significant factor that influences soil microbes. Drought, a probable consequence of grassland ecosystems in the future, has observed noticeable changes in richness, composition, and function of soil bacteria in North America and Australia but lesser changes in fungi (Ochoa-Hueso et al., 2018).

Fungal community changes are directly proportional to the precipitation gradient (Ochoa-Hueso et al., 2018; Wu et al., 2020). In Inner Mongolia, soil bacteria diversity significantly differs by habitat, while fungi taxa are less affected by declining precipitation. This suggests that bacteria are more sensitive than fungi, and the community structure of microbes is context-dependent (Wang et al., 2021).

Additionally, our results indicate that grazing intensity significantly changes fungi β diversity but does not affect the diversity of bacteria, which supports our hypothesis. Surface disturbance has an inconsistent effect on soil microbial community. Soil microbial communities can recover quickly following disturbance treatment events, and their diversity is not highly affected by grazing disturbance treatment due to their resilience (Comer and Perkins, 2021). Overall, our experiments suggest that climate factors significantly impact soil microbes more than grazing intensity, which is consistent with our hypothesis. A probable reason for this is that a relatively long grazing history on four steppes have conferred high ecosystem resilience to different grazing and gradually adapted to different grazing intensities. However, future studies are required to determine the effects of both grazing and climate change on soil microbes. Soil microbial reaction to environmental changes is often subject to temporal lags, given that their biogeochemical responses to various environmental changes often take time as the soil microorganisms adaption. Nonetheless, soil microorganisms can lead microbial reactions and metabolic interactions in instantaneous microscale conditions.

### 4.2 Interactive effects between climate factors and grazing for vegetation and soil physicochemical property

Livestock grazing is widely regarded as the most widespread and intensive land use on Earth, especially in northern China, which includes some of the most extensive remaining grasslands on the Eurasian steppe (Kemp et al., 2013). Grazing has multiple effects on soils, including physical disturbance, such as soil compaction, and chemical disturbance, such as nutrient cycling via animal dung, which can alter plant productivity and ecosystem function. Both climate factors and grazing intensity significantly affect plant and soil physicochemical properties, including aboveground biomass (AGB), richness, nitrate- and ammonium-N, soil microbial biomass carbon (MBC), and soil microbial biomass nitrogen (MBN).

A regional-scale study on a global scale has shown that livestock grazing has negative effects on aboveground biomass and belowground biomass (Díaz et al., 2007), similar to our findings under grazing intensity (**Supplementary Tables S1**, **S2**). Our study discovered that grazing mainly reduced AGB, possibly due to livestock preference for perennial bunchgrasses and rhizome grass (Semmartin et al., 2010). The grazing optimization hypothesis suggests moderate grazing increases aboveground biomass through compensatory vegetation growth. However, grazing decreased AGB at 10 sites (Li et al., 2020), suggesting that grazing effects on AGB have exceeded compensatory growth (Wang and Wesche, 2016). Global models of biomass for grazing showed that grazing has negative effect on aboveground biomass (decrease 23%) but positive effects on belowground biomass (increase 20%), with the effects compensated for by nearly equivalent (Milchunas and Lauenroth, 1993). However, our regional-scale study showed that grazing reduced AGB and BGB. Grazing affects the biomass of vegetation and biodiversity, while these changes are not synchronous. Our research has shown that grazing intensity significantly changes plant richness, which was determined by vegetation composition, soil environment (context-dependency of the environment), and biodiversity indices such as species richness and vegetation evenness (Melendez Gonzalez et al., 2019). Our results indicated that livestock grazing did not affect plant species’ evenness. However, they positively affected richness, resulting in unchanging species diversity (Shannon index). These results contradict previous studies suggesting that grazing increase plant diversity due to light limitation, reducing competition by circumjacent plants (Borer et al., 2014; Guo et al., 2023), as well as influencing soil nutrients or water availability at the same time (Eldridge and Delgado-Baquerizo, 2018). Meta-analysis in large-scale shows that plant richness and aboveground net primary productivity decreased but evenness and belowground net primary productivity increased under livestock grazing. The decrease in plant richness due to grazing may be due to the level of herbivory for diet preference, with selective effects on plant community composition (Wan et al., 2015). The alteration of plant evenness resulting from grazing may attribute to livestock’s paramount ingestion for aboveground biomass of constructive species, and providing chance for other plant species to have higher resource accessibility (Wan et al., 2015). Domestic livestock of grazing significantly affected the C and N dynamic circulation in grassland ecosystems (Wu et al., 2014). Global meta-analysis showed that livestock grazing significantly decreased belowground C and N pools in global grassland ecosystems. Light grazing benefits soil C and N sequestration, while other grazing (gradient moderate and heavy grazing) significantly reduced C and N reservoir (Zhou et al., 2017), as in our findings with different climate factors under grazing intensity (**Tables S1**, **S2**). Livestock grazing decreased soil C and N pools by planting ingestion, reducing aboveground plant biomass, and decreasing root elongation and biomass, which reduced C allocation to roots (McSherry and Ritchie, 2013). Specifically, above-belowground biomass may increase under light grazing, stimulating more photosynthate entering into belowground roots and fixing C, leading to more root biomass and root exudates. More root exudates can increase C accumulation and enhance soil N inputs (Zhang et al., 2015). However, moderate and heavy grazing sharply decreases soil C and N pools, which is attributed to higher grazing intensity removing more aboveground vegetation at semi-arid and arid ecoregions (Smith et al., 2012). Soil microbial biomass carbon and nitrogen decrease with the same trend as C and N under moderate and heavy grazing.

### 4.3 Pathway of vegetation and soil physicochemical property for soil microbes

Soil microbes are vital for biogeochemical cycles in the ecosystem. They interact with plants and soil properties, meanwhile can be influenced by climate factors and human activities. Studies showed that difference of diversity of soil microbes depend on soil and vegetation variables (Ma et al., 2021). Grassland ecosystems existed a strong and consistent connection with plant communities and soil microbes at different scales (Bai et al., 2022). Grazing can alter the soil microbes community and diversity by affecting plant growth and soil properties (soil C/N ratio, soil moisture, and so on) (Wang et al., 2022).

Plant diversity is crucial in distribution of soil bacterial communities. Our study showed that bacteria β diversity is indirectly affected by plant belowground biomass, associated with root properties such as root exudate release, root cell death, and litter biomass deposition (Ma et al., 2022). These factors influence the soil organic matter and contribute to soil carbon and nitrogen accumulation. Plant community, including biomass, community composition, and biodiversity, ultimately determines the quantity and quality of soil composition. The variation in plant communities can change microbes’ communities, while soil properties play essential roles in this process.

Our research found that soil C and N content were the driving factors for bacteria (such as soil organic carbon, nitrate-nitrogen, and so on). The soil nutrient content was reduced with grazing intensity increased, ultimately regulating the bacteria community. Interestingly, MAP significantly influenced the β diversity of fungi, which was indirectly correlated with soil properties such as nitrate-nitrogen and ammonium-nitrogen. Soil moisture changes are also strongly correlated with the composition of fungi, dominant taxa, and specific groups in our study.

In conclusion, soil microbes are critical engines for biogeochemical cycles and interact with plants and soil properties. Research has shown that diversity of soil microbes altered because of soil and vegetation variables. Plant community and soil properties are essential in regulating the microbial community, which is complex. Future research should focus on understanding how these factors interact and influence each other in ecosystems.

## 5 Conclusions

Soil microbes are small but essential components of grassland ecosystems. Our study has shown that climate factors, as well as grazing intensity, can affect soil microbes. Different climate factors can influence microbial communities in various ways, as different climate patterns can impact vegetation and soil properties. The pathways through which climate factors and grazing intensity impact soil microbial communities are diverse. Bacteria β diversity is influenced by vegetation and soil carbon and nitrogen, while fungi β diversity is mainly affected by soil nitrogen.

The effect of grazing types on soil microbes is more significant than grazing intensity. Grazing intensity primarily affects vegetation and soil properties rather than soil microbes. This means that the response of soil microbes to grazing intensity can have a time lag and be inconsistent. Our research provides an innovative and comprehensive perspective on how human activity and regional scale can alter soil microbial communities.

In conclusion, soil microbes are crucial components of grassland ecosystems, and our study demonstrates climate factors and grazing intensity can influence these microbial communities. Bacteria and fungi microbes respond differently to these factors, and grazing types can have a more significant effect than grazing intensity. This research provides new insights in human activities and grazing intensity can alter soil microbial communities.

## Data availability statement

The original contributions presented in the study are included in the article/**Supplementary Material**. Further inquiries can be directed to the corresponding author.

## Author contributions

YZ, NL and GY conceived the ideas and designed the study. BQ, BW, YW, ML, and YB collected the data. BQ and BW analyzed the data. BQ wrote and revised the draft. and YZ revised the manuscript. All authors contributed to the article and approved the submitted version.
