# Supplementary material for: Climate, not grazing, influences soil microbial diversity through changes in vegetation and abiotic factors on geographical patterns in the Eurasian steppe
Source: Front Plant Sci. 2023 Sep 6;14:1238077. doi: 10.3389/fpls.2023.1238077 (PMC10511900; doi:10.3389/fpls.2023.1238077)
Supplement: Supplementary file 1 [file DataSheet_1.docx]

Supplementary Material

**Climate, not grazing, affected soil microbial diversity by altering vegetation and abiotic factors on geographical patterns in the Eurasian steppe**

Bademuqiqige^1,2^, Bin Wei^1,2^, Yuqi Wei ^1,2^, Mohan Liu^1,2^, Yixian Bi^1,2^, Ruixuan Xu^1,2^, Lu Lian^1,2^, Nan Liu^1,2^, Gaowen Yang^1,2^, Yingjun Zhang^1,2*^

*** Correspondence:**E-mail: zhangyj@cau.edu.cn

# Supplementary Tables

**Table S1** Soil physicochemical characteristics in different grazing intensity in four grasslands

| Grassland | Intensity | pH | EC (%) | SMC (%) | NO_3_^-^-N (mg•kg^-1^) | NH_4_^+^-N (mg•kg^-1^) | TN(g•kg^-1^) | C/N | SOC (g•kg^-1^) | AP (mg•kg^-1^) |
| --- | --- | --- | --- | --- | --- | --- | --- | --- | --- | --- |
|  | CK | 7.89±0.03a | 223.23±15.19a | 22.26±0.79b | 1.61±0.15c | 9.8±1.36ab | 3.07±0.12b | 12.87±0.47a | 35.27±1.08b | 1.55±0.09a |
| AS | LG | 7.903±0.05a | 207.3±8.07a | 23.87±0.75ab | 1.97±0.27b | 8.43±0.76b | 3.61±0.16a | 11.73±0.15a | 36.73±1.36ab | 1.48±0.04a |
|  | MG | 7.867±0.03a | 201.97±10.43a | 25.06±0.86a | 2.36±0.23ab | 10.17±0.34ab | 3.38±0.06ab | 11.59±0.12ab | 34.02±0.49a | 0.97±0.17a |
|  | HG | 7.94±0.03a | 218.47±3.14a | 22.2±0.1b | 2.92±0.02a | 11.76±0.93a | 3.44±0.01a | 10.85±0.21b | 32.73±0.1a | 0.98±0.28a |
|  | CK | 6.9±0.3a | 183±49.58a | 0.37±0.02a | 4.11±0.57b | 6.44±0.88c | 3.89±0.43a | 10.56±0.37a | 5.29±1.01ab | 5.293±1.0142ab |
| MS | LG | 6.84±0.01a | 130.1±64.7ab | 0.33±0.03a | 4.43±0.32b | 12.8±0.73b | 3.98±0.36a | 10.65±0.27a | 3.98±0.61b | 3.983±0.6085b |
|  | MG | 6.8±0.06a | 109.83±74.5b | 0.33±0.005a | 4.61±0.32b | 16.53±2.45ab | 3.93±0.03a | 10.43±0.2a | 7.92±1.21a | 7.923±1.2142a |
|  | HG | 6.56±0.16a | 124.4±45.63ab | 0.37±0.02a | 6.79±0.36a | 19.36±0.61a | 3.94±0.08a | 10.35±0.06a | 4.92±0.47b | 4.921±0.4713b |
|  | CK | 7.86±0.05a | 134.23±22.84a | 15.37±0.75a | 0.45±1.42c | 2.58±0.51c | 1.93±0.2a | 24.98±1.84a | 42.39±0.52a | 4.87±0.75a |
| TS | LG | 6.88±0.05b | 55.75±15.81b | 15.5±0.1a | 1.51±3.24bc | 2.54±0.26c | 2.54±0.26a | 26.16±1.24a | 35.56±4.43a | 4.97±0.65a |
|  | MG | 6.92±0.03b | 96.24±25.49ab | 12.86±0.48b | 0.23±4.04ab | 6.99±0.87a | 1.71±0.05a | 24.68±2.16a | 38.94±3.44a | 5.56±0.79a |
|  | HG | 6.96±0.03b | 82.96±16.88ab | 14.35b0.58ab | 2.06ab2.87a | 4.6±0.13b | 1.65±0.05a | 17±2.51b | 18.86±4.99b | 2.72±0.32b |
|  | CK | 8.03±0.01b | 173.8±5.75b | 2.85±0.32a | 0.83±1.21b | 2.25±0.13b | 1.03±0.1a | 7.42±0.24a | 8.67±2.45a | 0.53±0.1a |
| DS | LG | 8.16±0.04a | 261.13±11.94a | 3.1±1.3a | 0.31±1.92ab | 3.27±0.48a | 0.98±0.04a | 7.96±0.56a | 6.13±0.28a | 0.84a0.3a |
|  | MG | 8.13±0.02a | 148.77±4.5c | 2.46±0.44a | 0.28±2.66a | 2.68±0.18ab | 0.98±0.17a | 7.56±0.68a | 5.45±0.41a | 0.62a0.02a |
|  | HG | 8.02±0.04b | 120.4±4.16d | 2.6±0.31a | 0.63±2.5a | 3.36±0.16a | 0.89±0.05a | 8.78±0.08a | 5.87±0.34a | 1.14a0.35a |

**Notes:** Lower case letters indicated significant differences at different treatments at the 0.05 level for a Tukey test. CK: no grazing, LG: Light grazing, MG: Moderate grazing, and HG: Heavy grazing; Alpine steppe (AS), mesic meadow steppe (MS), intermediate moisture typical steppe (TS), and dry desert steppe (DS); Soil water content (SWC), Soil total carbon (SOC), Soil ammonium (NH_4_^+^) and nitrate (NO_3_^-^), Available phosphorus (AP), Total nitrogen (TN)

**Table S2** Vegetation and microbes characteristic in different grazing intensity in four grasslands

| Grasslands | Intensity | AGB (g·m^-2^) | BGB (g·m^-2^) | Richness | Evenness | Shannon | MBC | MBN |
| --- | --- | --- | --- | --- | --- | --- | --- | --- |
|  | CK | 220.67±55.41a | 2255.57±276.86bc | 14.667±0.6667a | 0.828±0.0677a | 2.215±0.1479a | 816.22±15.01a | 90.35±6.46a |
| AS | LG | 200.97±13.54a | 3832.07bc788.73a | 11.333±0.6667a | 0.828±0.0707a | 2.001±0.1497a | 707.84±9.35ab | 78.09±2.02ab |
|  | MG | 196.15±15.5a | 4433.74a549.07a | 11.333±1.8559a | 0.905±0.0834a | 2.161±0.1673a | 673.24±3.73ab | 72.35±0.11ab |
|  | HG | 238.21±26.52a | 1933.11a468.13bc | 11.667±0.8819a | 0.87±0.0246a | 2.129±0.0144a | 618.46±21.08b | 51.26±15.74b |
|  | CK | 180.01±21.9a | 1413.16±243.95a | 14±0.58b | 0.77±0.01a | 2.03±0.06a | 1123.84±138.82a | 142.05±16.59a |
| MS | LG | 157.02±5.42ab | 1865.53±235.5a | 21.33±1.2b | 1.01±0.31a | 3.11±1.001a | 1113.66±93.86a | 107.1±7.47b |
|  | MG | 108.14±25.24b | 1589.24±219.68a | 22.67±3.38b | 1.58±0.73a | 4.67±1.87a | 887.89±79.71a | 99.98±5.29b |
|  | HG | 108.53±8.79b | 1974.81±92.29a | 33.67±3.76a | 1.18±0.34a | 4.14±1.2a | 916.14±36.3a | 79.72±6.92b |
|  | CK | 191.27±15.88a | 1040.62±103.25a | 6.67±0.67a | 0.79±0.03a | 1.49±0.06a | 525.48±14.67a | 226.57±28.49a |
| TS | LG | 234.43±55.09a | 1195.19±37.45a | 6.33±0.67a | 0.72±0.04b | 1.34±0.14b | 456.28±11.37b | 162.41±1.93b |
|  | MG | 175.24±5.03a | 704.88±106.86b | 6.67±0.33a | 0.78±0.01a | 1.48±0.06a | 388.96±23.8c | 119.35±14.04bc |
|  | HG | 66.03±16.36b | 590.52±80.34b | 7.33±0.33a | 0.83±0.001a | 1.66±0.04a | 253.53±24.77d | 69.82±5.34c |
|  | CK | 109.84±18.87a | 293.5±21.19a | 10.33±0.67ab | 0.81±0.06a | 1.9±0.12b | 93.32±4.38d | 24.38±2.09a |
| DS | LG | 79.86±11.42ab | 246.06±84.06ab | 8.33±0.67b | 0.83±0.11a | 1.75±0.03ab | 580.11±16.06a | 22.08a1.14a |
|  | MG | 69.16±12.96ab | 186.89±34.3ab | 11±0a | 0.87±0.23a | 2.1±0.06a | 264.42±20.99b | 15.09a2.63a |
|  | HG | 53.03±4.46b | 106.1±40.37b | 8.33±0.67b | 0.86±0.2a | 1.82±0.14a | 170.56±35.13c | 18.18a4.39a |

**Notes:** Lower case letters indicated significant differences at different treatments at the 0.05 level for a Tukey test. CK: no grazing, LG: Light grazing, MG: Moderate grazing, and HG: Heavy grazing. Alpine steppe (AS), mesic meadow steppe (MS), intermediate moisture typical steppe (TS), and dry desert steppe (DS); Aboveground biomass (AGB), Belowground biomass (BGB), microbial biomass carbon (MBC), and microbial biomass nitrogen (MBN).


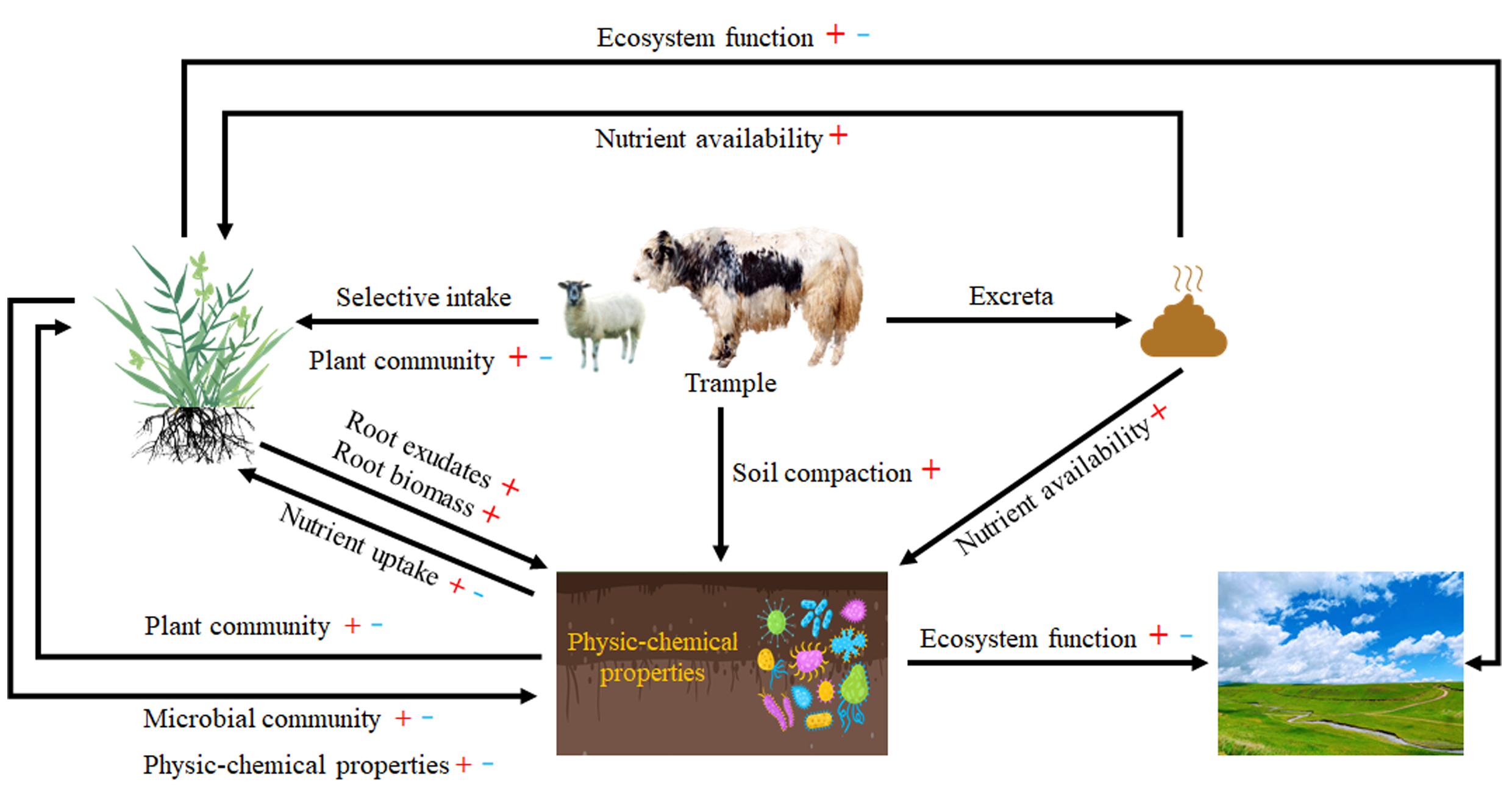


**Supplementary Figure 1.** Conceptual illustration showing the potential links among grazing, plant and soil microbial communities, and ecosystem function. “+” represents positive effects, and “-” represents negative effects.


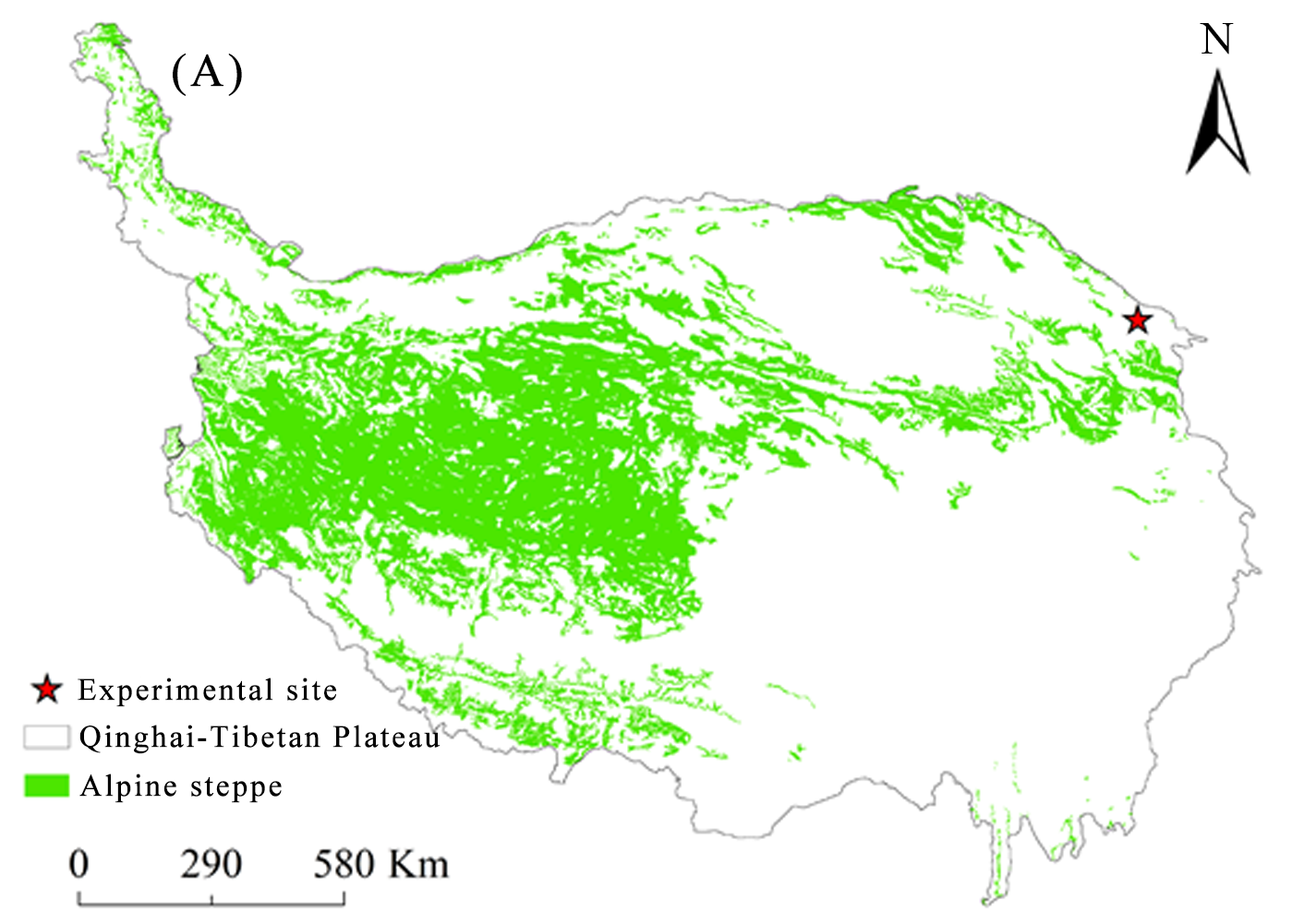


**Supplementary Figure 2.** Geographical location of the sampling sites in the present study.


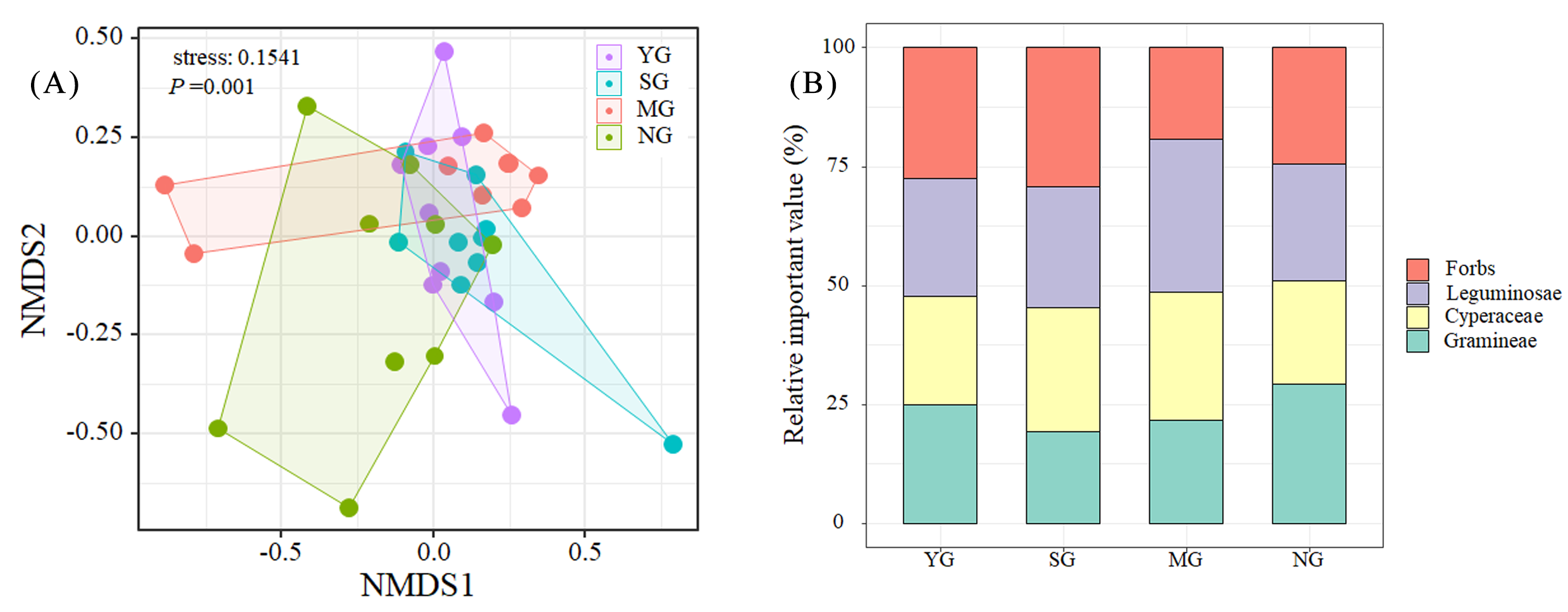


**Supplementary Figure 3.** Non-metric multidimensional scaling (NMDS) ordination of all sampling units indicating the relative differences in plants (A); Relative important values of plants under different herbivore assemblages (B). YG: Yak grazing; SG: Tibetan Sheep grazing; MG: Yak and Tibetan sheep mixed grazing; NG: No grazing.


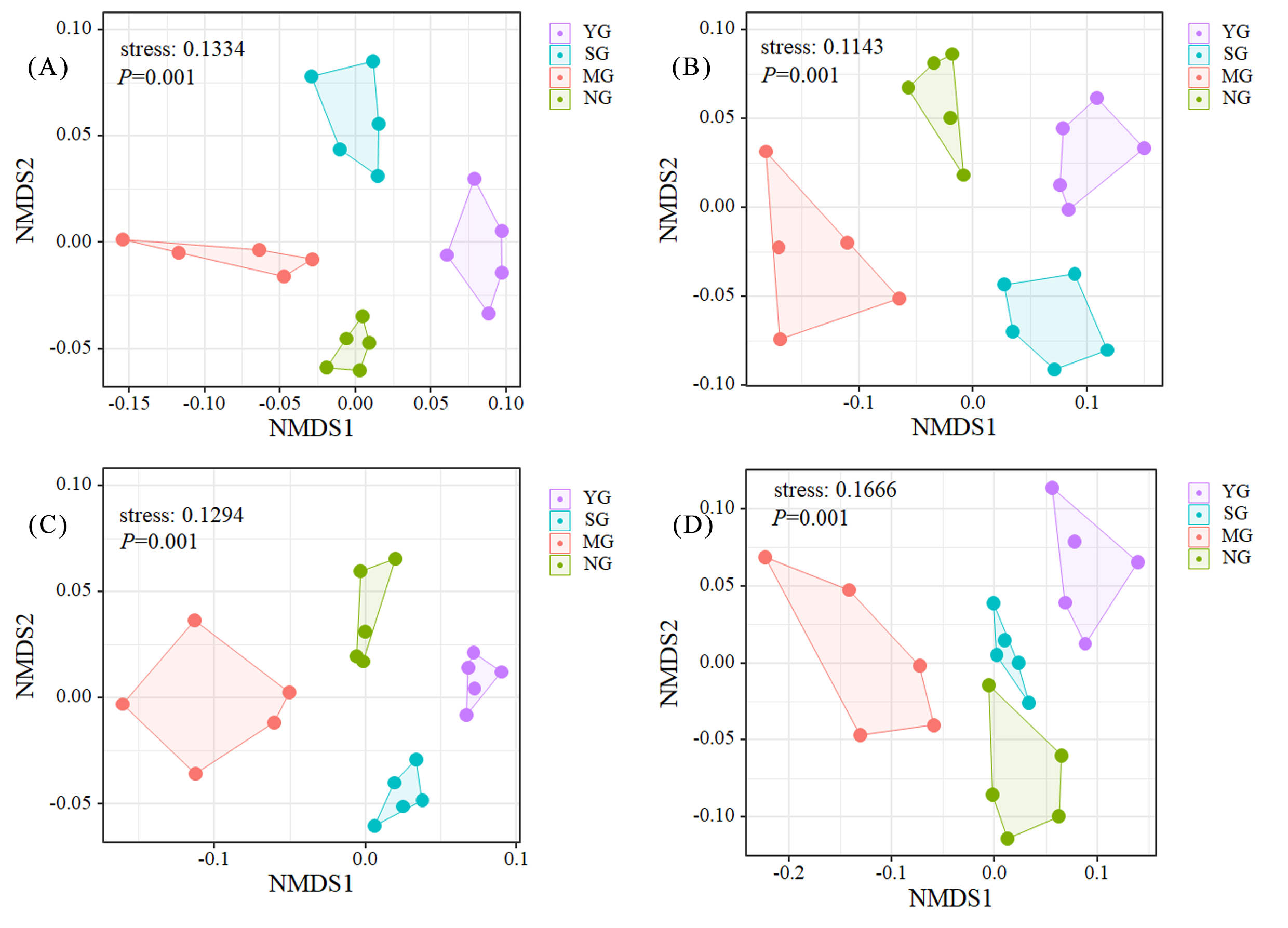


**Supplementary Figure 4.** Non-metric multidimensional scaling (NMDS) ordination of all sampling units indicating the relative differences in bacterial phyla Actinobacteria (A), Proteobacteria (B), Acidobacteria (C) and Planctomycetes (D) community compositions. YG: Yak grazing; SG: Tibetan Sheep grazing; MG: Yak and Tibetan sheep mixed grazing; NG: No grazing.


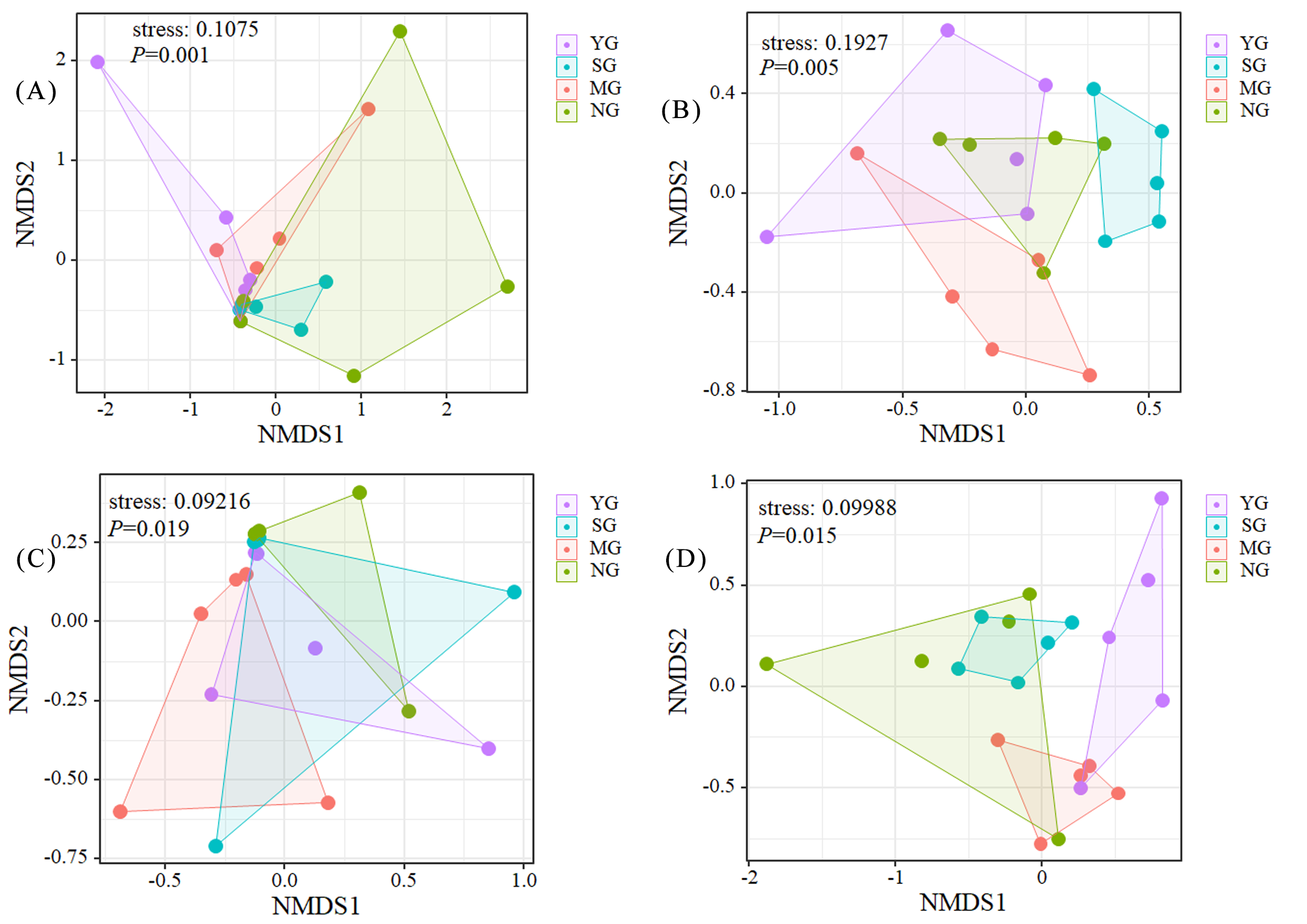


**Supplementary Figure 5.** Non-metric multidimensional scaling (NMDS) ordination of all sampling units indicating the relative differences in fungal families Clavicipitaceae (A), Nectriaceae (B), Pseudeurotiaceae (C) and Saccharomycetaceae (D) community compositions. YG: Yak grazing; SG: Tibetan Sheep grazing; MG: Yak and Tibetan sheep mixed grazing; NG: No grazing.


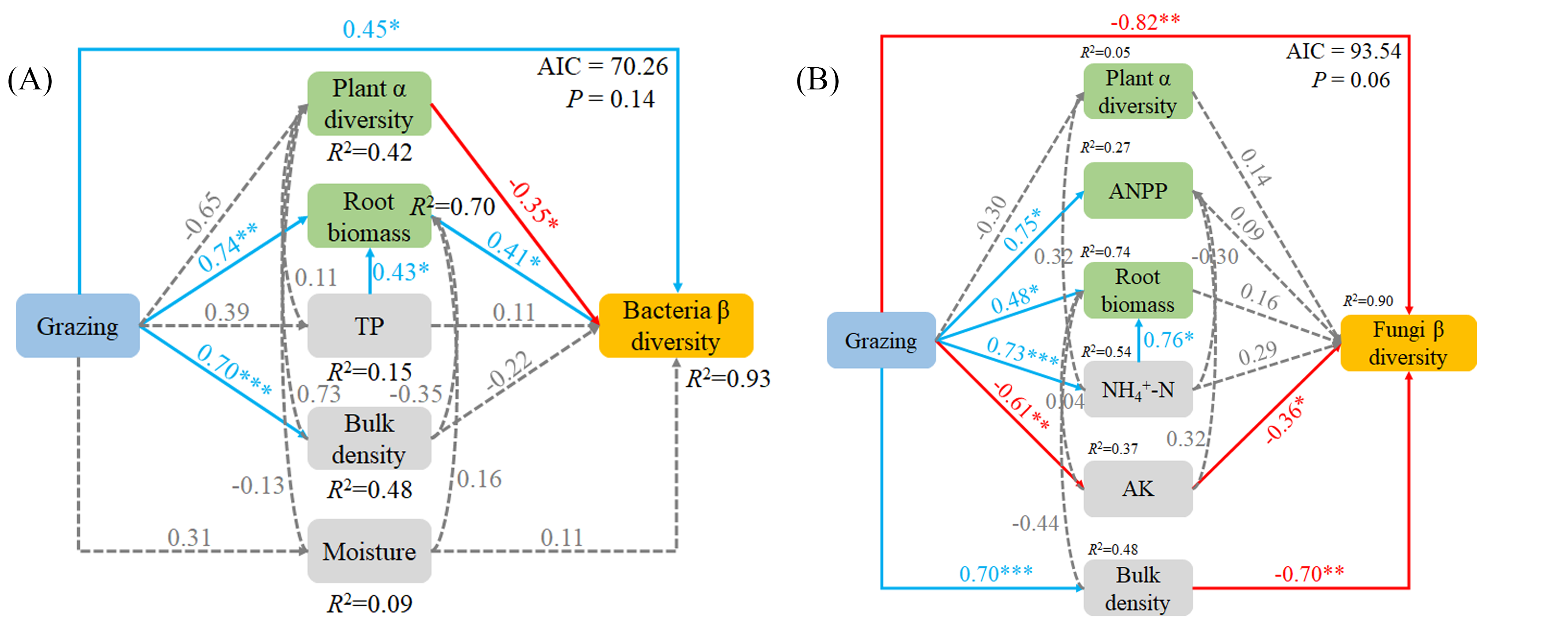


**Supplementary Figure 6.** Hypothetical causal model for structural equation modelling (SEM).

## Supplementary Tables

**Supplementary Table 1.** Relationships between soil microbial community and biotic and abiotic variables. Bold values represent significant relationships. TC: total soil carbon; TN: total soil nitrogen; C/N: carbon/nitrogen; TP: total soil phosphorus; NO3--N: soil nitrate; NH4+-N: soil ammonium; AP: soil available phosphorus; AK: soil available potassium; SBD: soil bulk density; ANPP: aboveground net primary productivity.

| Variables | Bacteria | | Fungi | |
| --- | --- | --- | --- | --- |
|  | *r*^2^ | *p* | *r*^2^ | *p* |
| TP (g·kg^-1^) | **0.356** | **0.022** | **0.388** | **0.012** |
| NO_3_^-^-N (mg·kg^-1^) | 0.171 | 0.210 | **0.357** | **0.025** |
| NH_4_^+^-N (mg·kg^-1^) | 0.165 | 0.227 | **0.530** | **0.005** |
| AP (mg·kg^-1^) | **0.661** | **0.001** | **/** | **/** |
| AK (mg·kg^-1^) | 0.076 | 0.522 | **0.360** | **0.037** |
| pH | 0.087 | 0.483 | 0.052 | 0.627 |
| Moisture (%) | **0.324** | **0.038** | 0.094 | 0.404 |
| SBD (g·cm^-3^) | **0.676** | **0.001** | **0.366** | **0.021** |
| Shoot biomass (g·m^-2^) | 0.012 | 0.916 | 0.110 | 0.333 |
| Root biomass (kg·m^-2^) | **0.347** | **0.032** | **0.322** | **0.038** |
| ANPP (g·m^-2^) | 0.088 | 0.442 | **0.301** | **0.046** |
| Plant α diversity | **0.516** | **0.003** | **0.306** | **0.047** |

# Supplementary Material

**Appendix S1.** Details of DNA extraction and high‐throughput sequencing methodology.

Soil DNA was extracted from 0.25 g using the HiPure Soil DNA Kit (Magen, Guangzhou, China) according to the manufacturer’s protocol. Twenty DNA extracts were obtained and then stored at -80°C for further analyses. DNA degradation and impurity were detected by 1% agarose gel electrophoresis, DNA purity was assessed by NanoDrop 2000 UV-vis Spectrophotometer (Thermo Fisher Scientific, Wilmington, DE, USA), and DNA concentration was determined on a Qubit 3.0 Flurometer (Thermo Fisher Scientific). The hypervariable V3-V4 region of the bacterial 16S rRNA gene was amplified with primer pairs 341F (5’-CCTACGGGNGGCWGCAG-3’) and 806R (5’-GGACTACHVGGGTWTCTAAT-3’) by an ABI GeneAmp® 9700 PCR thermocycler (ABI, Foster City, CA, USA). The fungal ITS1 region was amplified using the primers ITS1_F_KYO2 (50-TAGAGGAAGTAAAAGTCGTAA-30) and ITS86R (50-TTCAAAGATTCGATGATTCAC-30). HiSeq sequencing and PE250 sequencing strategy were used.
